# Emphysematous pyelonephritis in a young male Syrian patient: A rare case report

**DOI:** 10.1016/j.radcr.2022.05.093

**Published:** 2022-06-27

**Authors:** Maher Al-Hajjaj, Muhamad Kanjo

**Affiliations:** a Department of Urology, Aleppo University Hospital, Aleppo, Syria; b Department of Internal Medicine, Aleppo University Hospital, Aleppo, Syria

**Keywords:** Nephrectomy, Emphysematous pyelonephritis, Case report

## Abstract

Emphysematous pyelonephritis, a necrotizing kidney infection, is a rare condition with a poor prognosis. We present a case of 24-year-old male with fever, malaise, and left flank pain. Computed tomography of abdominal and pelvis showed left emphysematous pyelonephritis. Treatment included IV antibiotics. A left nephrectomy was necessitated for definitive treatment. Clinical course after surgery was unremarkable. In conclusion, prompt diagnosis of emphysematous pyelonephritis is necessary to reduce morbidity and mortality. In some cases, nephrectomy is the definite treatment.

## Introduction

Emphysematous pyelonephritis (EPN) is an acute severe necrotizing infection of the renal parenchyma and its surrounding tissue and leads to gas production in the renal parenchyma, collecting system, or perirenal tissue [1].

CT scan is mandatory not only to establish the diagnosis but also for follow- up and to decide the best line of treatment. Early diagnosis and management may facilitate renal preservation but delay in diagnosis and treatment contributes to high morbidity and mortality rates even today [2].

We describe a rare case of EPN in a 24-year-old male with poorly controlled, insulin-dependent type 2 DM.

This case report was prepared in accordance with the SCARE guidelines [3].

## Case presentation

A 24-year-old male with a history of poorly controlled, insulin-dependent type 2 DM presented to the ED with fever, malaise, left flank pain, and dysuria for 3 days. No other important past medical or surgical history. On exam, vital signs were: blood pressure 90/60 mm/Hg, pulse 140 bpm, and temperature 39.8°C. Physical examination showed delayed response to commands with mild disorientation. He had abdominal tenderness especially in the left flank. Examination of other systems showed no important findings.

His laboratories are shown in Table 1. Urinalysis demonstrated proteinuria (+1), hematuria (+2), and glycosuria (+3). Urine microscopy showed the presence of red blood cells 30-40/HPF, pus cells of >60/HPF.

**Table 1:** Laboratory values at admission.

WBC	Hgb	PLT	Cr	Urea	Glu	PH	Na	K
23 × 10^9^/ml	11.2 gm/dl	350 × 10^5^/mcl	1.8 mm/dl	157 mg/dl	320 mg/dl	7.18	140 mEq/L	5.5 mEq/L

**Fig. 1:**
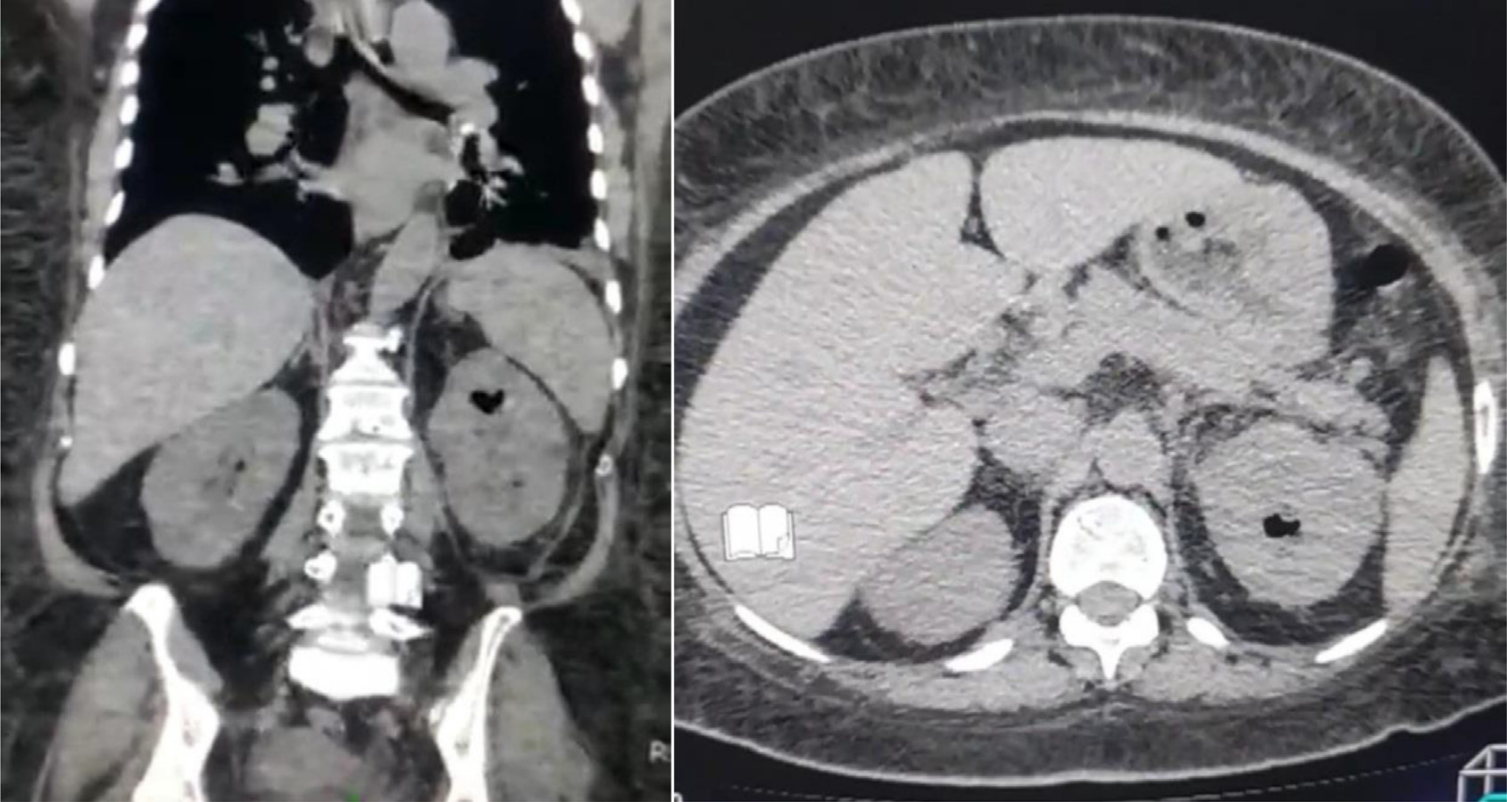
Computed tomography (coronal and axial views) showing gas in the left kidney.

**Fig. 2:**
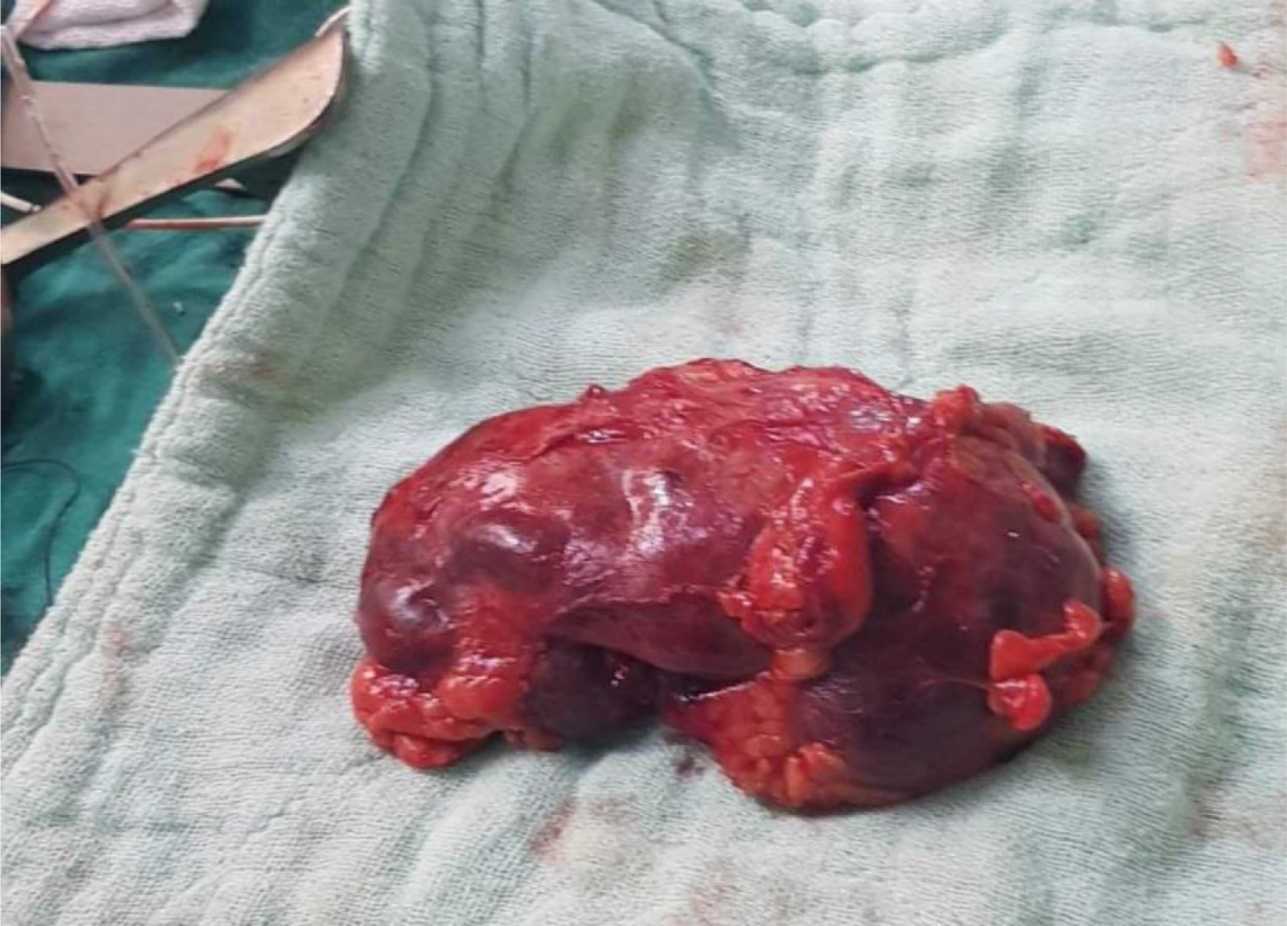
Left kidney after resection.

Immediately, we started with fluid resuscitation with normal saline and we added insulin therapy to achieve target glycemic control. We sent urine specimen for culture. Abdominal and pelvis computed tomography (CT) scan revealed gas inside the left kidney (Fig. 1). We started with intravenous antibiotics with meropenem and levofloxacin. The diagnosis was left EPN. Two days later, the patient did no improve as expected. Instead, he had lower blood pressure, high temperature, and severe left flank pain. After taking patient consent, the patient was admitted to surgical room. Through the subcostal approach, we performed left simple nephrectomy (Fig. 2). We needed 75 minutes to finish. There were no problems during surgery. After surgery, the patient spent 2 days in the intensive care unit (ICU). Postsurgery, we monitored wbc, Hgb, plt, creatinine, urea, glucose, and PH. They are shown in Table 2. Urine culture revealed heavy growth of *E. coli* which was sensitive to the meropenem and levofloxacin. Third day after surgery, we transferred the patient to the ward. He spent just one night and he was discharged on regular urological clinic visits. Follow-up for 6 months showed complete remission with normal blood tests including renal function and glucose level.

**Table 2:** Laboratory values at intensive care unit.

In ICU	WBC	Hgb	PLT	Cr	Urea	Glu	PH	Na	K
Day 1	16 × 10^5^/ml	10.6 × 10^5^gr/dl	317 × 10^5^/mcl	1.1 mg/dl	80 mg/dl	156 mg/dl	7.32	142 mEq/L	4.1 mEq/L
Day 2	11 × 10^5^/ml	10.8 × 10^5^gr/dl	320 × 10^5^/mcl	1 mm/dl	45 mg/dl	140 mg/dl	7.37	138 mEq/L	4 mEq/L

## Discussion

EPN is an acute severe necrotizing infection of the renal parenchyma and its surrounding tissue and leads to gas production in the renal parenchyma, collecting system, or perirenal tissue [1].

Diabetes mellitus is the single most common associated factor. Up to 95% of patients with EPN have underlying uncontrolled diabetes mellitus. Other reported factors associated with the development of EPN are drug abuse, neurogenic bladder, alcoholism and anatomic anomaly [4].

Plain abdominal radiography may show abnormal gas in the renal bed or collecting system. CT is the preferred diagnostic modality because of its high sensitivity and ability to assess the extent of gas patterns [1].

Treatment of EPN can be conservative with or without minor interventions or nephrectomy.

The mortality rate of the disease ranges between 11% and 54% [5].

To our knowledge, this case is very rare because of the age of our patient (24 years old). Our case was a young male patient with poorly controlled DM type 2 diagnosed with EPN. The medical history and physical examination led to suspect EPN. We started first with fluid resuscitation. Empiric antibiotics were administrated via intravenous. CT scan of the abdomen and pelvis was the gold standard to confirm the diagnosis. Based on the clinical state of our patient, he underwent to simple left nephrectomy. The course after surgery was unremarkable. With strict control of glucose levels, he got full remission. Follow-up showed no complications.

## Conclusion

EPN is an emergency case. Even with young ages, it has a high mortality. Urologists should react immediately to deal with the case and give the suitable management for the patient.

## Patient consent

Written consent for publication of this case was obtained from the patient and is available upon request. Additionally, the patient received and approved this manuscript.
